# *Penicillium* sp. as an organism that degrades endosulfan and reduces its genotoxic effects

**DOI:** 10.1186/2193-1801-3-536

**Published:** 2014-09-17

**Authors:** Mariana Romero-Aguilar, Efrain Tovar-Sánchez, Enrique Sánchez-Salinas, Patricia Mussali-Galante, Juan Carlos Sánchez-Meza, María Luisa Castrejón-Godínez, Edgar Dantán-González, Miguel Ángel Trujillo-Vera, Ma Laura Ortiz-Hernández

**Affiliations:** Universidad Autónoma del Estado de Morelos, Av. Universidad 1001, Col. Chamilpa, C. P. 62209 Cuernavaca, Mor, México; Facultad de Química, Paseo Colón esquina Paseo Tollocan, Universidad Autónoma del Estado de México, S/N. C.P. 50120 Toluca, México; Servicio Nacional de Sanidad, Inocuidad y Calidad Agroalimentaria, Carretera Federal Cuernavaca-Cuautla No. 8534 Col. El Progreso Jiutepec, Morelos, C.P. 62550 México

**Keywords:** Endosulfan, Biodegradation, Genotoxicity

## Abstract

Endosulfan is an organochloride and persistent pesticide that has caused concern because of its impact in the environment and its toxicity to and bioaccumulation in living organisms. In this study, we isolated an endosulfan-degrading fungus from the activated sludge from an industrial wastewater treatment plant. Through repetitive enrichment and successive subculture in media containing endosulfan as the sole carbon source, a fungus designated CHE 23 was isolated. Based on a phylogenetic analysis, strain CHE 23 was assigned to the genus *Penicillium* sp. In a mineral salt medium with 50 mg/l endosulfan as the sole source carbon, CHE 23 removed the added endosulfan in a period of six days. To verify the decrease in endosulfan toxicity due to the activity of the fungus, we performed genotoxicity tests trough the single cell gel electrophoresis assay or comet assay, with *Eisenia fetida* as the bioindicator species. This organism was exposed to the supernatants of the culture of the fungus and endosulfan. Our results indicated that the genotoxicity of endosulfan was completely reduced due the activity of this fungus. These results suggest that the *Penicillium* sp. CHE 23 strain can be used to degrade endosulfan residues and/or for water and soil bioremediation processes without causing toxicity problems, which are probably due to the generation of no-toxic metabolites during biodegradation.

## Background

Endosulfan (*6,7,8,9,10,10-hexachloro-1,5,5a, 6,9,9a-hexahydro-6, −9 methano-2,3,4-benzo-dioxathiepin-3-oxide*) is a cyclodiene organochlorine insecticide that possesses a relatively broad spectrum of activity. It has been used worldwide in a variety of crops, such as pear, broccoli, squash, potatoes, cereals, coffee, cotton, and oilseeds (Elsaid et al. 2010; Kalyani et al. 2010; Singh and Singh 2011; Hatipoglu et al. 2008).

Commercial-grade endosulfan is a mixture of two stereoisomers, namely α- and β -endosulfan, at a ratio of 7:3, respectively (Singh and Singh 2011; Kataoka et al. 2010). These stereoisomers are widely distributed in the environment and can be found in soil and sediments over long distances from the direct source of the application. The endosulfan half-life in soils is estimated to range from 60 to 800 days; however, its half-life in groundwater and sediments may increase up to six years. This chemical can be incorporated into the atmosphere through agricultural application or induced-temperature volatilization. Because of its widespread use and distribution in the environment, endosulfan contaminates soils, groundwater, and sediments (Castillo et al. 2011; Kalyani et al. 2010; Kataoka et al. 2010; Singh and Singh 2011; Weber et al. 2009; Hussain et al. 2007a, [b]; Kumar and Philip 2007).

In general, endosulfan isomers are converted through oxidation to endosulfan sulfate (more toxic than endosulfan) or by hydrolysis to endosulfan diol, a less toxic metabolite. In the environment, endosulfan sulfate is the most frequently detected residue, particularly in soils and plant and animal tissues (Castillo et al. 2011; Kataoka et al. 2010; Kumar and Philip 2007).

Several studies have demonstrated the toxicity of endosulfan. This chemical has been found to be extremely toxic to fish (Velasco-Santamaría et al. 2011; Ballesteros et al. 2007), is recognized as an endocrine disruptor in amphibians (Kataoka et al. 2010; Hussain et al. 2007a), and is highly toxic to aquatic invertebrates (Chaudhuri et al. 1999). Endosulfan exposure has been shown to affect both the reproductive and endocrine systems of experimental animals and humans (Dalsenter et al. 2003). Moreover, it has been reported that endosulfan causes neurotoxicity (Silva de Assis et al. 2011; Zhenquan and Hara 2007). In general, one of the main mechanisms underlying endosulfan toxicity is the induction of several genotoxic insults, including chromosomal aberrations in peripheral lymphocytes in field workers (Carbonell et al. 1995; Rupa et al. 1991; Rupa et al. 1989), single strand breaks in freshwater fishes (Sharma et al. 2007; Pandey et al. 2006), and micronucleus induction in tadpole erythrocytes (Lajmanovicha et al. 2005). Additionally, exposure of the earthworm *Eisenia fetida* to different doses of endosulfan produced DNA strand breaks (Liu et al. 2009).

Wessel et al. (2007) found genotoxic damage in embryos of *Crassostrea gigas* exposed to increasing concentrations of endosulfan that resulted in DNA chain breakage. Similarly, Bajpayee et al. (2013) demonstrated that chain breaks in Chinese hamster ovary cells and human lymphocytes is dependent on the endosulfan concentration and the exposure time. Neuparth et al. (2006) reported that endosulfan causes chromosomal damage in goldfish (*Sparus aurata*) found in water contaminated from agricultural soil runoff. Sharma et al. (2007) determined that endosulfan causes double and single DNA breaks, adduct formation, and DNA-DNA and DNA-protein cross-linking without leading to death in *Mystus vittatus*.

The genotoxic effects of endosulfan in humans have not been widely studied. Using comet assays, Bajpayee et al. (2006) found an increase in genetic damage in human lymphocytes exposed to endosulfan, endosulfan lactone, and endosulfan sulfate. Occupational exposure was studied by Topé and Rogers (2009), who described the effects in humans occupationally exposed to a mixture of pesticides, including endosulfan. Endosulfan genotoxicity was also analyzed in HepG2 cells, and breaks of the DNA chain were observed (Yuquan et al. 2000).

Taking into account the extent of use and the high occurrence of endosulfan in the environment, as well as the hazardous risk of endosulfan exposure to humans and wildlife, it is necessary to develop both *in situ* and *ex situ* treatment techniques that promote endosulfan degradation in the environment. The use of organisms isolated from contaminated sites allows the generation of biologically efficient and low-cost methods for the treatment of xenobiotic compounds. The detoxification of endosulfan through biological means is receiving serious attention as an alternative to the existing methods, such as incineration and landfill (Siddique et al. 2003). An advantage of these methods is that endosulfan can be used as the sole source of carbon and/or sulfur during the biodegradation process (Kumar and Philip 2006; Sutherland et al. 2000; Guerin 1999).

Endosulfan degradation by microorganisms has been studied mainly with bacteria isolated from soils contaminated with pesticides over long periods of time. Some bacterial species whose removal has been demostrated are *Klebsiella pneumonia*, *Pseudomonas spinosa*, *Pseudomonas aeruginosa*, *Burkholderia cepacia*, *Stenotrophomonas maltophilia*, *Rhodococcus erythropolis*, *Achromobacter xylosoxidans*, *Mycobacterium* sp*.*, *Arthrobacter* sp*.*, *Pseudomonas* sp*.*, *Bordetella* sp*.*, and *Pseudomonas* sp. (Singh and Singh 2011; Bajaj et al. 2010; Goswami et al. 2009; Hussain et al. 2007a; Kumar and Philip 2007; Lee et al. 2006; Weir et al. 2006; Kwon et al. 2002; Sutherland et al. 2002).

In contrast, studies of endosulfan degradation by filamentous fungal organisms are scarce. Fungal organisms have advantages over bacterial strains, e.g., the fungi enzymes of the lignocellulolytic complex have been related to the degradation of various xenobiotic pollutants, including pesticides. The disadvantages of some fungal strains include the growth and degradation times. For example, Bhalerao and Puranik (2007) achieved endosulfan degradation using *Aspergillus niger*, but the complete mineralization process required a period of 12 days. *Phanerochaete chrysosporium*, *Aspergillus terreus*, *Aspergillus terricola*, *Chaetosartorya stromatoides*, *Mortierella* sp*.*, *Trametes hirsute*, and *Mucor thermohyalospora* are able to remove 50 to 90% of endosulfan over a period of 12 to 28 days (Kamei et al. 2011; Elsaid et al. 2010; Kataoka et al. 2010; Hussain et al. 2007b; Siddique et al. 2003; Shetty et al. 2000; Kullman and Matsumura 1996).

After applying a pesticide degradation process using microorganisms, it is necessary to analyze the decrease in the pesticide concentration in the culture medium and to assess the decrease in toxicity. This assessment can be accomplished through the use of short-term tests, which provide information on the level of DNA damage caused by a genotoxin. In this context, the alkaline single-cell gel electrophoresis assay, which is also known as the comet assay, is a sensitive, reliable method for detecting alkali-labile and delayed repair sites, which are measured as DNA single-strand breaks, in eukaryotic individual cells. The comet assay is considered as an early biomarker of a biological effect and is widely used to assess DNA damage both *in vivo* and *in vitro* (Mussali-Galante et al. 2013; Rojas et al. 1996; Valverde et al. 1997).

In the present work, we isolated a fungus from an industrial wastewater treatment plant and tested its ability to degrade endosulfan. Furthermore, genotoxicity tests based on the comet assay using a bioindicator organism (*Eisenia fetida*) were conducted to analyze if its genotoxicity decreases by the isolated fungi activity.

## Results

### Identification of CHE 23 strain

**Figure 1:**
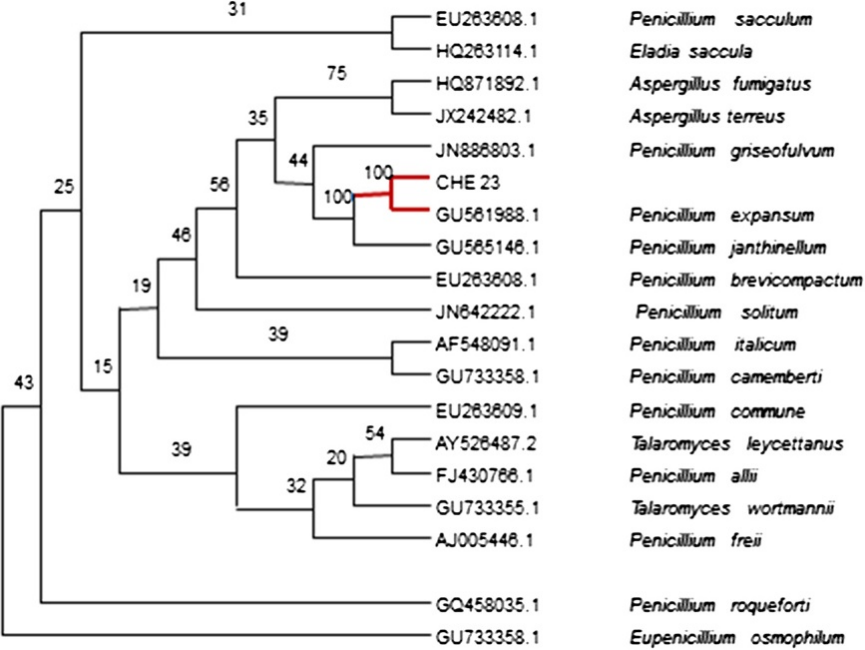
**Phylogenetic tree based on the sequences of the 18S rRNA region of the CHE23 fungus, isolated from waste sludge.** The numbers at the branches indicate bootstrap values, with nine clades with bootstrap values from 15 to 100%.

We performed a BLAST-n search with the CHE 23 strain rRNA 18S (GenBank accession Number KJ503282) sequence to find similarities with reported sequences. The 744-bp RNA 18S rDNA query sequence showed 99% sequence identity with *Penicillium expansum* (GU561988.1). A 744-bp sequence from the 18S rDNA region the CHE 23 strain rRNA 18S (GenBank accession Number KJ503282) was used to construct a phylogeny with 18 other sequences retrieved by BLASTn. The percentages of the query coverage analyses ranged up to 100%. Sample results from this analysis include 100% identity with *Penicillium expansum* (GU561988.1) and with *Penicillium janthinellum* (GU565146.1), and 75% identity was found with *Aspergillus fumigatus* (HQ871892.1) and *Aspergillus terreus* (JX242482.1), to name a few of the results (Figure 1).

### Growth kinetics of the CHE 23 strain

**Figure 2:**
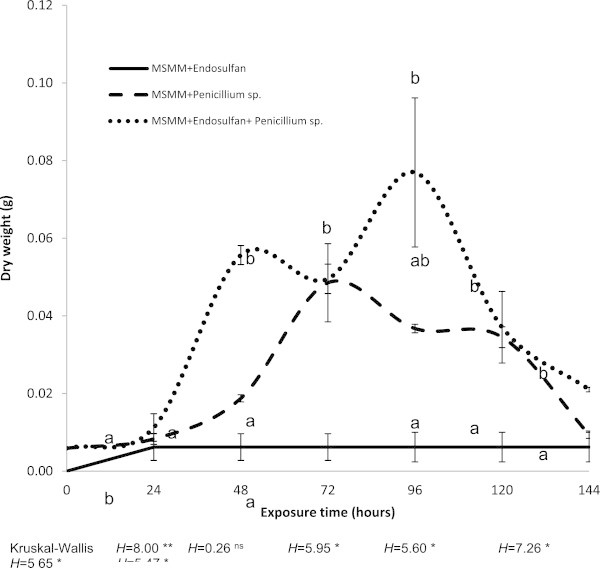
**Fungal growth measuring every 24 hours for 144 hour.** Different letters show significant differences at * = P < 0.05, ** = P < 0.01 (Kruscal-Wallis significant difference test).

The growth of *Penicillium* sp. CHE 23 with the different treatments is shown in Figure 2. We observed biomass production after 24 h of exposure to endosulfan; however, after 72 h, the growth decreased, and a higher biomass production was observed at 96 h (mean ± SD, 0.077 ± 0.033 g). This behavior suggests diauxic growth, which means that the strain uses the MMSM nutrients and then the pesticide as its carbon source. This behavior was not observed in the treatment without pesticide (Figure 2). Higher biomass production was observed in the culture media enriched with endosulfan.

### Removal of endosulfan from the culture medium by *Penicillium*sp

Table 1 shows the concentrations of endosulfan at the beginning and the end of the kinetics experiments. *Penicillium* sp. was able to decrease the initial endosulfan concentration to 2.9 mg/l (94.87%) in MMSM after 144 h. A removal of 15.31% of the initial endosulfan was observed in the uninoculated controls. The comparison of the endosulfan removal achieved by the fungus through a variance analysis indicated statistically significant differences between the treatments (*F*_2,15_ = 241.75, *P* < 0.001). Tukey’s test showed that *Penicillium* sp. removed significantly a higher proportion of endosulfan (P < 0.05), compared with the treatment control. We should mention that the full details of endosulfan removal rate in MMSM will be presented in another paper.

**Table 1:** **Endosulfan concentrations at the beginning and after 144 hours of** ***Penicillium*** **sp. culture**

	0 hours		144 hours	
	(mg/l)	Endosulfan remainder (%)	(mg/l)	Endosulfan remainder (%)
MMSM + Endosulfan + *Penicillium* sp.	56.73 ± 0.55a	100	2.91 ± 0.85c	5.12
MMSM + Endosulfan	56.73 ± 0.55a	100	48.04 ± 3.05b	84.68

Different letters show significant differences at *P < 0.001* (Tukey’s honestly significant difference test).

(*F*_2,15_ = 241.75, *P* < 0.001).

### Toxicity tests

#### Acute toxicity

The mortality of earthworms exposed to the culture media obtained from the different treatments, was measured. When earthworms were exposed to *Penicillium* sp. culture medium, they showed no mortality after 48 h of exposure, but a weight loss of 8%, likely due to starvation, was observed (Table 2). In contrast, the organisms exposed to MMSM + endosulfan treatment showed 100% mortality after 48 h of exposure. All organisms that were exposed only to the MMSM medium remained alive. Regarding weight loss, the results are consistent with the observed mortality, as the average weight loss of earthworms exposed to *Penicillium* sp. culture media was lower in comparison with the weight loss of exposed organisms to endosulfan, without contact with the fungus. These results suggest that the endosulfan toxicity was decreased by the fungus activity through the pesticide’s removal/transformation in culture medium.

**Table 2:** **Acute toxicity on earthworms exposed to culture supernatant of** ***Penicillium*** **sp. in MMSM and endosulfan**

Treatment	Earthworm expose during 24 hours		Earthworm expose during 48 hours	
	Weight loss (g)	Mortality (%)	Weight loss (g)	Mortality (%)
MMSM + Endosulfan + *Penicillium* sp.(t_0_)	1.08 ± 0.62	23	1.31 ± 0.70	100
MMSM + Endosulfan + *Penicillium* sp.(t_f_)	0.19 ± 0.02	0	0.47 ± 0.11	0
MMSM + Endosulfan (t_0_)	1.08 ± 0.62	23	1.31 ± 0.70	100
MMSM + Endosulfan (t_f_)	1.02 ± 0.01	17	1.24 ± 0.07	100
MMSM + *Penicillium* sp. (t_0_)	0.08 ± 0.02	0	0.22 ± 0.08	0
MMSM + *Penicillium* sp. (t_f_)	0.09 ± 0.04	0	0.15 ± 0.07	0

We used culture media of *Penicillium* sp. at the beginning (t_0_) and after 144 hours of culture (t_f_).

#### Genotoxicity

**Figure 3:**
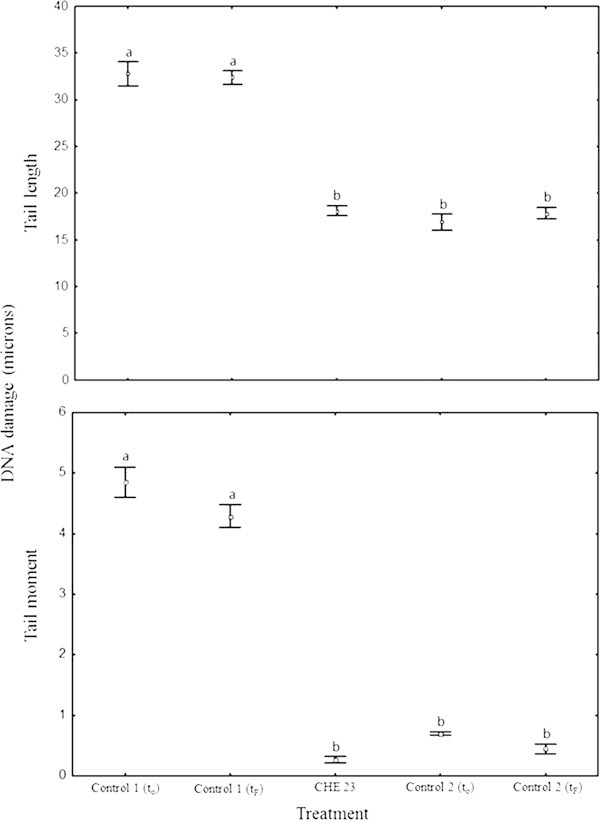
**DNA damage in** ***Eisenia fetida*** **celomocytes (tail length and tail moment).** Different letters show significant differences at *P* < 0.05. Control 1: MMSM + Endosulfan (t_0_) initial time and final time (t_f_), Control 2: MMSM + *Penicillium* sp. (t_0_) initial time and final time (t_f_), CHE 23: MMSM + Endosulfan + *Penicillium* sp.

The DNA damage levels observed in *Eisenia fetida* organisms exposed to endosulfan are shown in Figure 3. In general, the analysis of variance (ANOVA) detected that the treatment with *Penicillium* sp. strain had a significant effect on DNA damage levels (*F*_4,140_ = 88.614, *P* < 0.001). In general, Tukey’s test showed that the controls (MMSM + endosulfan [t_0_ and t_f_]) exhibited the highest levels of DNA damage levels (tail length), and no differences were found between the initial and final times. In contrast, the treatments MMSM + *Penicillium* sp. initial time (t_0_) and final time (t_f_), presented the lowest levels of DNA damage. The treatment MMSM + endosulfan + *Penicillium* sp. had a DNA damage similar to the MMSM + *Penicillium* sp. treatment only after de 144 hours of culture with the fungus. A similar pattern was detected when DNA damage levels were quantified as tail moment (*F*_4,140_ = 239.48, *P* < 0.001).

At the beginning of the kinetics experiment, the earthworm exposed to the culture supernatant of *Penicillium* sp. (treatment MMSM + endosulfan + *Penicillium* sp.) showed an average DNA damage (tail length) of (mean ± SD) 33.09 ± 5.67 microns. When the coelomic fluid cells where exposed to the fungus culture media after 144 h of culture, they showed an average DNA damage of 18.11 ± 0.53 microns, and this reduction was statistically significant (Tukey test, *P* < 0.05). In contrast, the exposure of the earthworm coelomocytes to MMSM + endosulfan treatment resulted in significantly higher DNA damage at the end of the experiment (32.37 ± 0.076) than the previous treatment. Furthermore, the cells exposed to endosulfan coelomic fluid without endosulfan and in the presence of the fungus (MMSM + *Penicillium* sp.) showed a tail length of 18.08 ± 1.34 microns, and no significant differences were observed between the initial and final times. These last two treatments were considered as controls. The DNA damage values found with MMSN + endosulfan + *Penicillium* sp. treatment at the end of the experiment were equivalent to those obtained without endosulfan, suggesting that the fungus exhibits detoxifying activity against the pesticide.

As shown in Figure 3, the DNA damage was significantly reduced when endosulfan was removed from the culture medium by *Penicillium* sp. The OTM results were consistent with the tail length results.

## Discussion

The environmental concerns and human health issues caused by the indiscriminate use of chemical pesticides, makes necessary the development of environmentally and economically viable strategies for the degradation or endosulfan elimination. This approach would permit the mitigation of the negative impacts of this pesticide on the environment and the risks for human health. In this work, we isolated a fungus from an activated sludge of an industrial wastewater treatment plant and subsequently this fungus was identified as *Penicillium* sp.

Under the experimental conditions used of this study, *Penicillium* sp*.* strain CHE 23 was capable of removing 94.87% of the initial concentration of endosulfan added to the culture medium after six days, which is a shorter period than that reported by previous studies. These data demonstrated the greater removal efficiency of the isolated organism compared with those reported in the literature. For example, Bhalerao and Puranik (2007) reported the complete mineralization of endosulfan by *Aspergillus niger* isolated from contaminated soil with an endosulfan initial concentration of 350 mg/l; the variables used to measure this mineralization were changes in the pH conditions and increases in the concentration of CO_2_ over a period of 12 days. *Phanerochaete chrysosporium* was able to degrade 90% of the initial concentration of endosulfan over a period of 14 days, resulting in the production of endosulfan sulfate and endosulfan diol metabolites (Kullman and Matsumura 1996). Kamei et al. (2011) reported that the species *Trametes hirsuta* degrades 90% of endosulfan and 70% of endosulfan sulfate over a period of 10 days to produce endosulfan lactone and endosulfan hydroxy-ether as the final metabolites. Other findings that are in accordance with our results, are those reported by Shetty et al. (2000), who reported that *Mucor thermohyalospora* degrades α-endosulfan and β-endosulfan (70% and 50%, respectively) over a period of 20 days to produce endosulfan sulfate and endosulfan diol. Siddique et al. (2003) used *Fusarium ventricosum* to obtain degradation percentages of 91.1% for the α isomer and 89.9% for the β isomer after a 15-day period. Hussain et al. (2007b) also reported a 90% degradation percentage for both isomers over a 10-day period with *Aspergillus terreus*, *Aspergillus terricola*, *Chaetosartorya stromatoides* after 12 days of incubation and identified the final products as endosulfan and ether diol endosulfan. In addition, *Mortierella* sp. was found to be capable of degrading more than 70% and 50% of α- and β-endosulfan, respectively, after 28 days to generate endosulfan ether (Kataoka et al. 2010).

The same effect was observed with *Trametes hirsute* and *Aspergillus sydoni*, which obtained the endosulfan degradation of 90% and 95% after a period of 10 to 18 days, respectively. In addition, 90% and 97% of the α-and β-endosulfan metabolites were degraded, respectively, to obtain endosulfan sulfate, endosulfan diol, endosulfan ether, and endosulfan lactone (Goswami et al. 2009; Kamei et al. 2011).

In this work, the production of fungal biomass was measured. A statistically significant increase in growth was observed with the addition of endosulfan to the culture medium as the sole carbon source, suggesting that this pesticide is used as a source of carbon by the fungus. This behavior is important since this fungus has the potential to be used in the endosulfan wastes elimination or in contaminated soils and groundwater remediation. Therefore, we should continue to investigate this fungus strain because the treatment time may decrease compared with those obtained with other strains that were also isolated from environmental samples.

In the present study, after having measured the ability of endosulfan removal by *Penicillium* sp., we conducted studies of acute toxicity and genotoxicity using *Eisenia fetida* as an indicator species. According to the results of acute toxicity, as measured in weight loss and mortality, the results show a decrease in toxicity due to the endosulfan removal/transforming by the fungus activity.

For genotoxicity studies, after exposure of *Eisenia fetida* to culture media, we used the comet assay under alkaline conditions (pH > 13). This assay is able to detect DNA damage (i.e., single-strand breakage or other lesions) such as alkali-labile sites, DNA cross-links, and incomplete excision repair events. It offers considerable advantages over the other cytogenetic methods such as chromosome aberrations, sister chromatid exchanges, and the micronucleus test used to detect DNA damage, because for the comet assay, the cells need not to be mitotically active (Buschini et al. 2003, Sharma et al. 2007).

The genotoxic potential of endosulfan has been reported by previous studies. Liu et al. (2009) determined the toxicity of endosulfan in *Eisenia fetida* after its exposure to soil containing endosulfan at a final concentration of 10 mg/kg and found that the greatest damage could be measured after 28 days of exposure; these results indicate the genotoxic potential of this pesticide. Sharma et al. (2007) tested various non-lethal concentrations of endosulfan to assess the genetic damage induced in cells from different tissues of *Mystus vittatus* using the comet assay. Liu et al. (2009) determined the genotoxic damage induced by different endosulfan concentrations on *Trifolium repens* L. (white clover) and *Eisenia fetida* (earthworm).

In this work, we found that the endosulfan toxicity after being exposed to *Penicillium* sp., fell almost 100%. These results suggest the pesticide transformation to non-toxic metabolites, or probably due endosulfan sulfate is also degraded by the fungus. These findings are consistent with the results found in the endosulfan removal from the culture medium, also the behavior of the fungus growth in the MMSM supplemented with this compound.

Several authors have described the degradation pathway by fungal activity. Kamei et al. (2011) tested endosulfan degradation with *Trametes hirsuta* that was capable of degrading 90% endosulfan and endosulfan sulfate, a toxic metabolite resulting from oxidation of endosulfan that is equally persistent to, or more persistent than the parent endosulfan isomer. This fungus degrades endosulfan sulfate by two pathways: the hydrolytic way producing endosulfan diol, which decreases its toxicity approximately three orders of magnitude and is subsequently transformed to endosulfan ether and endosulfan lactone (Wan et al. 2005). The second way has not been described and leading to the production of dimethylene endosulfan. Kataoka and Takagi (2013) found very similar results. In addition, the degradation of endosulfan without forming endosulfan sulfate has been reported in earlier studies (Kwon et al. 2002). Many bacteria capable of degrading endosulfan as well as its toxic metabolites endosulfan sulfate had been reported (Singh and Singh 2011).

In the present study, the metabolites resulting from the degradation were not identified or measured; thus, it was not possible to identify the fungal metabolic pathway underlying the removal of endosulfan. However, according to the resulting chromatograms in a test performed on GC-MS (data not shown), no endosulfan sulfate metabolites were detected throughout the experiment, suggesting their transformation into less toxic metabolites or the possible complete mineralization of the compound.

In this paper, we have shown that *Penicillium* sp. has the ability to degrade endosulfan and probably endosulfan sulfate. In addition, we show evidence that the resulting solutions do not result in biological effects (toxicity and genotoxicity) on *Eisenia fetida* organisms. According to these findings, we can mention that this fungus has the potential to be used in the treatment of endosulfan’s wastes and/or remediation of contaminated soil and water.

## Conclusions

*Penicillium* sp. strain CHE 23 has the capacity to remove endosulfan from de culture media. Unlike previous studies, the present study showed that the isolated strain of *Penicillium* sp. is able to decrease 100% of the genotoxic effects of endosulfan. Although the secondary metabolites were not determined in this work, the reduction of the biological effects (genotoxicity) indicates that the more toxic endosulfan metabolites were either not generated or also degraded. Thus, the *Penicillium* sp. strain isolated from the activated sludge of an industrial wastewater treatment plant has the potential to be used in waste treatment approaches to eliminate the presence and toxic effects of endosulfan.

## Methods

### Reagents

For the enrichment of activated sludge, commercial-grade endosulfan (Tridane 350) was used. For the analysis of the growth kinetics and degradation, technical-grade endosulfan was purchased from Chemservice (99% purity). Reactive-grade ethyl acetate was used as a solvent for pesticide extraction, and HPLC-grade hexane from Mallinckrodt Baker Inc. (Phillipsburg, NJ, USA) was used to inject the samples into the gas chromatograph. All of the other chemicals were of reagent grade and were obtained from J.T. Baker (Mexico City, Mexico).

### Culture media

A mineral salt medium (MSM) was used for the isolation and acclimation stage, and the composition of the medium was previously described by Yañez-Ocampo et al. (2009): 0.82 g/l K_2_HPO_4_, 0.19 g/l KH_2_PO_4_, 0.20 g/l MgSO_4_·7H_2_O, 2.0 g/l KNO_3_, 0.99 g/l (NH_4_)_2_SO_4_, and a 2 ml/l trace element solution, which was composed of 2.8 g/l H_3_BO_3_, 2.55 g/l MnSO_4_·H_2_O, 0.17 g/l CuSO_4_·5H_2_O, 2.43 g/l CoCl_2_·6H_2_O, and 0.25 g/l ZnSO_4_·7H_2_O, pH 7 ± 0.05. The carbon source was endosulfan at a final concentration of 150 mg/l. To conduct the experiments to analyze the fungus growth kinetics and endosulfan removal, a modified MSM (MMSM) was used; the composition of the MMSM was 1 g/l (NH_4_)_2_SO_4_, 0.5 g/l MgSO_4_·7H_2_O, 0.875 g/l KH_2_PO_4_, 0.125 g/l K_2_HPO_4_, 0.1 g/l CaCl_2_·H_2_O, 0.1 g/l NaCl, 0.0001 g/l FeSO_4_, 0.05 g/l MnCl_2_·4H_2_O, and 0.5 g/l casein peptone as an additional nitrogen source. To obtain the fungal biomass and use it as inoculum in the experiments, MMSM with 0.1% glucose was used.

### Enrichment and isolation of microorganisms

Microorganisms capable of degrading endosulfan were isolated from the activated sludge from an industrial wastewater treatment plant that receives wastewater from a pesticide industry.

A total of 200 ml of the activated sludge were collected in a 500-ml Erlenmeyer flask and transported to the laboratory at 4°C. To allow the microorganisms to acclimatize, the sludge was enriched with weekly addition of 150 mg/l commercial-grade endosulfan, and the sample was incubated at 30°C and shaken at 150 rpm; this process was continued for a period of five weeks.

After five weeks of acclimation, 1 ml of the sludge was transferred into a 150-ml Erlenmeyer flask with 80 ml of MSM and endosulfan as the sole carbon source at a final concentration of 150 mg/l. The flask was incubated for a week under the same conditions listed above. A volume of 5 ml of the resulting culture with 50 ml of MSM was used to inoculate four flasks, and the flasks were incubated for a week with shaking under the above-mentioned conditions. The microorganisms that grew in these cultures were plated on Potato Dextrose Agar plates (PDA, BD Difco, Becton Dickinson and Company) containing endosulfan at a concentration of 50 mg/l, and the plates were incubated at 30°C for 72 h. Well-separated colonies were selected and transferred repeatedly on the same solid medium to obtain pure cultures.

Through this procedure, we isolated 26 strains that were growth in MMSM with 50 mg/l endosulfan. To select the strains that grew best with endosulfan as the sole carbon source and that exhibited the capacity for endosulfan degradation, preliminary assays were conducted. The strain registered as CHE 23 was selected because of it exhibited both of these abilities (data not shown).

### Molecular identification of CHE 23

The molecular identification of the fungal strain CHE 23 was performed using the molecular marker rRNA 18S. The genomic DNA was extracted from an enriched culture following the instructions provided with the Ultraclean Microbial DNA Isolation kit (MoBio Cat. 12800–100). The PCR amplification was performed in a 20-μl reaction volume containing 2.5 U of Taq DNA polymerase (Fermentas Life Sciences), a 10× dilution of the manufacturer’s buffer (Fermentas Life Sciences), 200 μM of each deoxynucleoside triphosphate (dNTP), 20 pM of primers SS-1 F (5′TTAGCATGGAATATRRATAGGA3′) and 18SR (5′ATTGCATGCYCTATTCCCCA3′), and 50 ng of genomic DNA.

The reaction conditions were as follows: initial denaturation at 94°C for 3 min, 30 amplification cycles of denaturation at 94°C for 1 min, annealing at 48°C for 1 min, and primer extension at 72°C for 1 min, and a final extension at 72°C for 10 min. The PCR amplifications were conducted using a Thermo-Hybaid PCR thermal cycler (Thermo Fisher Scientific USA). Aliquots of the PCR products (5 μl) were analyzed in 1% (w/v) agarose gels through horizontal gel electrophoresis. The DNA was visualized by UV excitation after staining with ethidium bromide (0.5 mg/l). The PCR products were purified using the QIAquick Gel Extraction kit (Qiagen, Valencia, CA, USA). The 18S rRNA nucleotide sequence was determined through PCR-direct sequencing performed by the Institute of Biotechnology of the National Autonomous University of Mexico. The phylogenetic analysis of the 18S rRNA gene sequences was performed using the NCBI BLAST program. The phylogenetic trees were inferred using the neighbor-joining method, and bootstrap analyses were performed. The evolutionary distances were computed using the Maximum Composite Likelihood method.

### Preparation of inoculum

To obtain the fungal biomass, mycelia of the strain, which was previously grown on PDA solid medium, was inoculated into a 250-ml Erlenmeyer flask containing 100 ml of MMSM with 1 g/l glucose and 50 mg/l endosulfan. The inoculation was made with the addition of five circles, each of which had a diameter of 1 cm. The flask was incubated for 48 h at 30°C and 150 rpm. The resulting culture was used to inoculate flasks for the analysis of the growth kinetics of the strain and endosulfan degradation, as described below.

### Growth kinetics of CHE 23

Fifty-milliliter Erlenmeyer flasks with 20 ml of MMSM media without glucose were inoculated with 5 ml of the culture obtained from the inoculum preparation, which corresponds to 0.03 g ± 0.002 g biomass (dry weight). The flasks were incubated for 144 h at 30°C and 150 rpm. The following treatments were established in triplicate: MMSM with 50 mg/l endosulfan and the CHE 23 strain; MMSM and endosulfan at a final concentration of 50 mg/l without inoculum, and MMSM and inoculum without endosulfan. The last two treatments were considered controls. Every 24 h, the biomass production of the CHE 23 strain was measured based on its dry weight: the culture was filtered, washed with distilled water, and dried at 100°C for 24 h. The dry weight of the fungus was recorded and plotted as a function of time. At the beginning and end of the experiment, the concentration of the pesticide was quantified, and acute toxicity tests and genotoxicity assays were conducted, as described below.

### Endosulfan degradation tests using the CHE 23 strain

One-hundred-and-fifty-milliliter Erlenmeyer flasks with 50 ml of the MMSM media without glucose were inoculated with 5 ml of the culture obtained from the inoculum preparation, which corresponds to 0.03 g ± 0.002 g biomass (dry weight). The flasks were incubated for 144 h at 30°C and 150 rpm. The treatments were similar to those described in the previous section. The pesticide concentration was determined at 0 and 144 h.

### Analytical methods

At the beginning and the end of the experiments used to analyze the growth kinetics, 1-ml sub-samples were collected from each treatment for endosulfan analysis and placed in glass tubes. These sub-samples were extracted three times with equal volumes of ethyl acetate as the extracting reagent. The mixture was homogenized for 1 min by vortexing. The ethyl acetate with residual endosulfan was filtered, dried with anhydrous sodium sulfate, and filtered through glass-fiber paper (Whatman GF/B). This operation was conducted sequentially, and the filtrates were mixed. The filtrate was evaporated to dryness and resolved in 50 μl of HPLC-grade hexane for analysis.

The amount of pesticide was quantified on a gas chromatograph Varian 450 equipped with two ECD detectors with split-splitless Varian 1177 injector systems. An Agilent CP-Sil 5 CB silica capillary column with 100% dimethylpolysiloxane as the non-polar phase (15 mx, diameter 0.25 mm, film thickness 0.25 m) was used. The carrier gas was helium (purity 99.99%), and the flow rate was maintained constant at 1.3 ml min^−1^. The injector and detector temperature was maintained at 260°C. The oven temperature was programmed as follows: 130°C for 5 min, raised to 160°C at a rate of 5°C min^−1^, maintained at 160°C for 20 min, increased to 200°C at a rate of 5°C min^−1^, and maintained at 200°C for 15 min. The sample volume injected was 2 μl. Analytical-grade endosulfan (99%) was used as the standard for the computation of the residual concentration of endosulfan in the broth culture. The retention time of the respective peak was compared using the standard.

### Obtaining *Eisenia fetida*organisms

The earthworms (*Eisenia fetida*) used for the acute toxicity tests and comet assays were obtained from a vermicomposting center in Central Mexico. The worms were transferred to the facilities of the University of the State of Morelos, Mexico, where they were acclimatized for three weeks in a substrate rich in organic matter. Adult organisms with a well-developed clitellum and an average weight of 270 mg were used.

### Acute toxicity assays

The toxicity of endosulfan was evaluated by a modification of OECD guideline No. 207 (1998). Adult individuals of *Eisenia fetida* were used for the acute toxicity tests. These individuals presented a well-developed clitellum and an average weight of 270 mg. For these assays, we used culture supernatants obtained from the growth of the CHE 23 strain and endosulfan degradation kinetics experiments described above. Ten earthworms were exposed during 24 and 48 hours to 10 ml of the culture supernatant obtained after 144 hours of the growth of CHE 23 strain using the filter-paper contact test. This procedure was performed in triplicate for each treatment. The weight loss and mortality of the organisms were evaluated. The treatments used in this experiment were MMSM + endosulfan + CHE 23, MMSM + endosulfan, and MMSM + CHE 23 without pesticide.

### Genotoxicity test using the single-cell gel electrophoresis or comet assay

#### Slide preparation

Earthworms that were previously exposed for 12 h to the treatments in the acute toxicity tests were used for the genotoxicity tests using the comet assay. Ten organisms of *Eisenia fetida* were used for each treatment, and these organisms were placed individually in a Petri dish with 1 ml of phosphate buffer saline (1× PBS). Three cuts were made with a scalpel to each organism, and the organisms were allowed to stand for 30 s to obtain the coelomic fluid. Fifteen microliters from the resulting solution (mixture of coelomic fluid and phosphate buffer saline) were collected and placed in an Eppendorf tube with 75 μl of 0.5% low-melting point agarose (LMA). Seventy-five microliters of this mixture were placed on glass slides previously coated with 75 μl of 1% agarose. The slides were cooled at 4°C for 5 min. After the agarose gelled, 75 μl of 1% LMA agarose was added. The slides were immersed in lysis solution (14.6 g/70 ml NaCl, 0.12 g/70 ml Tris, 3.72 g/70 ml EDTA 3.72, 0.8 g/70 ml NaOH, 10 ml of dimethyl sulfoxide, and 1 ml of Triton X-100, pH 10) for 2 h at 4°C.

#### Electrophoresis

The slides were maintained for 20 min in an electrophoresis chamber (Power Pac 3000 BioRad) with electrophoresis and unwinding buffer at pH 13 (30 ml of 10 N NaOH and 5 ml of 200 mM EDTA). Subsequently, electrophoresis was performed under the following conditions: 20–25 V, 300 mA, and 4°C for 5 min. The slides were neutralized in 0.4 N Tris base buffer at pH 7.5 for three 5-min cycles and then dehydrated in 100% ethanol for 10 min.

#### Slide examination

Prior to examination, the slides were stained with 50 μl of 1× ethidium bromide. One hundred cells per slide were randomly scored using an image analysis system attached to an epifluorescent microscope (Carl Zeiss, Axiostar Plus H-BO-100) equipped with a 515- to 560-nm excitation filter and a 590-nm barrier filter. All of the steps were conducted in dim light to prevent additional nonspecific DNA breakage. The comet images were captured, and the Comet IV software (Perceptive Instruments) was employed to measure various comet parameters. The parameter used to quantify the extent of DNA damage was the olive tail movement (OTM), which is the product of the distance between the center of gravity of the head and the center of gravity of the tail and the percentage of tail DNA (Liu et al. 2009). Additionally, the comet tail length (DNA migration) was recorded.

### Statistical analysis

All of the statistical analyses were performed using the STATISTICA V.8 software from STAT Soft Inc. (USA, 2007). Analysis of variance (Kruskal-Wallis) was used to test the effect of the treatments (control 1, control 2, and CHE treatment) on *Penicillium* sp. (g) biomass production with different exposure times (0, 24, 48, 72, 96, 120, and 144 h) (Zar 2010). Additionally, a multiple comparison analysis test (Tukey) was used to determine the significance of the differences between the mean *Penicillium* sp. biomass values among the treatments.

For the evaluation of DNA damage, we used the Shapiro-Wilk “*W*” test, which is used to probe the normality of the results (Zar 2010). Our results show that the *W* test was not significant for any of the treatments (*P* > 0.05). Thus, we accepted the hypothesis that the data presented a normal distribution. We performed one-way ANOVA (model I fixed effects, Zar 2010) to detect whether the treatments had an effect on coelomocyte DNA damage (microns). Additionally, a multiple comparison analysis test (Tukey) was conducted to determine the significance of the differences between the mean DNA damage values (tail length and tail moment) among the treatments (Zar 2010).

In addition, one-way ANOVA was conducted to determine the effect of endosulfan treatment on the residual endosulfan concentration. A multiple comparison analysis test (Tukey) was conducted to determine the significance of the differences in the mean endosulfan concentrations among the treatments (Zar 2010).

## Abbreviations

ANOVA: Analysis of variance
DNA: Desoxiribonucleic acid
HPLC: High performance liquid chromatography
MSM: Mineral salt medium
MMSM: Modified MSM
PDA: Potato dextrose agar
rRNA: Ribosomal ribonucleic acid
dNTP: Deoxinucleoside triphosphate
PBS: Phosphate buffer saline
LMA: Low-melting point agarose
OTM: Olive tail movement.
